# Methodological Study to Investigate the Potential of Ultrasound-Based Elastography and Texture as Biomarkers to Monitor Liver Tumors

**DOI:** 10.3390/diagnostics10100811

**Published:** 2020-10-13

**Authors:** Salma Moalla, Charly Girot, Stéphanie Franchi-Abella, Samy Ammari, Corinne Balleyguier, Nathalie Lassau, Stéphanie Pitre-Champagnat

**Affiliations:** 1 Department of Diagnostic Imaging, Gustave Roussy, 114 Rue Edourad Vaillant, 94840 Villejuif, France; Samy.AMMARI@gustaveroussy.fr (S.A.); Corinne.BALLEYGUIER@gustaveroussy.fr (C.B.); nathalie.lassau@gustaveroussy.fr (N.L.); 2 Université Paris-Saclay, CEA, CNRS, Inserm, BioMaps Gustave Roussy, 94805 Villejuif, France; charly.girot@universite-paris-saclay.fr (C.G.); stephanie.franchi@aphp.fr (S.F.-A.); stephanie.pitre@u-psud.fr (S.P.-C.); 3 Department of Paediatric radiology, Hôpital Bicêtre, 78 Rue du Général Leclerc, 94270 Le Kremlin-Bicêtre, France

**Keywords:** 2D SWE, liver, characterization, elastography

## Abstract

Aims and objectives: In order to evaluate the responses of hepatic lesions to treatment in terms of tissue stiffness and heterogeneity, this work investigated the robustness of 2D shear-wave elastography (2D SWE) stiffness measurements and texture analyses in vitro and in vivo in terms of repeatability and variability. Methods and materials: A multioperator (*n* = 5) study was performed with an ultrasonic elastography device on two sets of phantoms. For the first set of phantoms, 10 measurements for each of the eight inclusions were performed by each observer, whereas the second set of phantoms was used to evaluate the influence of depth on the stiffness measurements. Variability of the stiffness measurements was evaluated in vivo on 10 healthy livers, with 10 measurements for each hepatic segment. Texture analyses were performed in B-mode, obtaining elastography images for every hepatic segment. Results: Stiffness measurements were influenced by depth, particularly when exceeding 7 cm. In vivo measurements demonstrated that measurements of segments I, VII, and VIII were less reliable, mainly due to their deeper locations. The protocols used were more flexible in terms of acquisition setup and probe placement than those currently used with Fibroscan^®^. For texture analysis on the B-mode images, 12 features showed low variability regardless of the evaluated hepatic segment. On elastogram, only two features showed low variability, but not in every segment. Conclusion: We demonstrated the robustness of two methodologies for the quantification of liver stiffness and heterogeneity. Further clinical studies should evaluate whether these techniques can assess tumor responses to treatment and, therefore, have the potential to be used as imaging biomarkers.

## 1. Introduction

Numerous new anticancer molecules recently emerged, such as antiangiogenic therapies, targeted therapies, and immunotherapies. Each molecule has its own impact on tumoral tissue. In addition to a possible decrease in size, which is well described in the literature with cytotoxic therapies, numerous changes in tumor characteristics are possible, such as partial or total angiogenesis decrease, necrosis, or lymphocyte influx into the tumor. These different outcomes make evaluation of tumor responses difficult. The evaluation of treatment response was initially based on tumor size evaluation (WHO criteria, RECIST 1.0, 1.1). These criteria, which are still used as clinical references, may not reflect accurate treatment responses since, in many situations, favorable responses to treatment were observed without decreases in size [1,2,3,4]. Under the effect of the new molecules, modifications of the composition, the cellularity, and/or the vascularization of tumors are possible. It is, therefore, easy to imagine that lesions can become more homogeneous or completely heterogeneous, or present modification of tumor stiffness. The development of new techniques to quantify and evaluate tumor responses is now regarded as an important issue.

Several new criteria were proposed to assess tumor responses in situations where Response evaluation criteria in solid tumors (RECIST) is faulty, such as the Choi criteria developed for gastro-intestinal stromal tumor (GIST), in particular, based not only on size decrease but also on density changes of the lesion. In the same field, studies conducted on the use of dynamic contrast- enhanced ultrasound (DCE-US) in the evaluation of tumor responses validated a new response biomarker that is particularly useful in the evaluation of antiangiogenic treatments [5].

Ultrasound-based elastography (UBE) methods are now widely available at low cost and have the advantage of no radiation. They are currently of great interest with a growing community, receiving attention in recent years for noninvasive assessment of tissue mechanical properties [6]. Different types of ultrasound-based elastography were developed and validated by the European Federation of Societies for Ultrasound in Medicine and Biology (EFSUMB) [7] such as strain elastography (SE), strain-rate imaging (SRI), acoustic radiation force impulse imaging (ARFI), transient elastography (TE), and shear-wave elastography (SWE). Stiffness modifications in tissues was evaluated and demonstrated to be useful for liver and nonliver indications [7] based on qualitative and quantitative information [8].

According to the World Federation for Ultrasound in Medicine and Biology (WFSUMB) [8] recommendations, ARFI and shear-wave imaging (SWI) techniques can be used to assess the severity of liver fibrosis in patients with chronic viral hepatitis, best demonstrated in patients with hepatitis C. Impulse elastography 1D, ARFI, and SWI are more accurate in detecting cirrhosis than significant fibrosis.

In recent years, several teams investigated the possibility of characterizing liver lesions on the basis of their stiffness, therefore enabling them to distinguish benign tumors from cancers [9,10] and to differentiate between different malignant lesions [11,12].

Using ultrasound-based elastography and texture analysis to assess possible modifications of tumor stiffness with treatment is an interesting approach. However, this approach only makes sense if stiffness measurements are reliable over the whole liver and not restricted to the right lobe, as reported in the literature, and if texture analysis on B-mode and SWI images of the liver is reliable regarding finding potential imaging biomarkers. As for SWI exploration, these features must be reliable over the different hepatic segments. 

To our knowledge, there are no studies or recommendations regarding the use of ultrasound-based elastography or texture analysis to evaluate the therapeutic responses of liver lesions in oncology via exploration of all hepatic segments. Thus, the purpose of our work was to evaluate the robustness of UBE and texture analyses in this field through the assessment of reproducibility, repeatability, and variability in vitro on phantoms and in vivo on healthy livers. 

## 2. Materials and Methods

### 2.1. Study Design and Data Analysis Methods 

Stiffness measurements based on high-frequency shear-wave elastography were performed using the ultrasonography machine Aplio i900 and the convex probe PVI-475BX, (Canon Medical Systems, Otawara-shi, Japan). The main acquisition parameters were as follows: Frame rate = 1; precision = 4; aplipure = 1; Dynamic Range = 70 dB; general frequency = 4 MHz. Stiffness measurements were directly reported and acquired images were saved in Digital Imaging and Communication in Medicine (DICOM) format for texture analysis processing. Stiffness measurements were characterized in terms of reproducibility with a coefficient of variation (CV) defined as CV = σ/μ, where σ is the standard deviation and μ is the mean of the corresponding parameters. All statistical analyses were performed using RStudio (RStudio Inc., Boston, MA, USA) software. A *p*-value threshold of 0.05 was considered statistically significant.

### 2.2. Ultrasonic-Based Elastography Phantoms 

**Figure 1:**
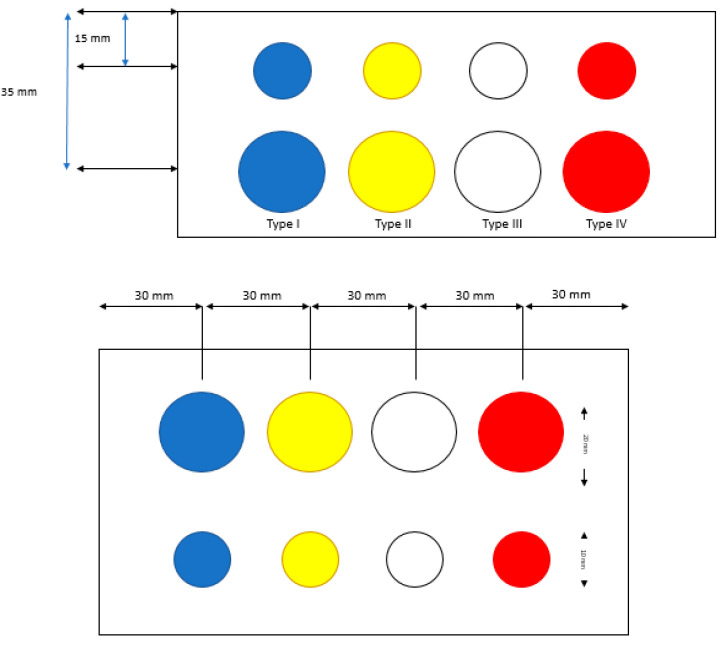
Elastography phantom 049 “Elasticity Phantom—Spherical target map” (CIRS, Norfolk, VA): Inclusions with diameters of 10 and 20 mm, at 15 and 35 mm deep, with different stiffness values for each pair. From left to right: Type I, 8 kPa; Type II, 14 kPa; Type III, 45 kPa; and Type IV, 80 kPa. Top: Front view; bottom: Upper view.

**Figure 2:**
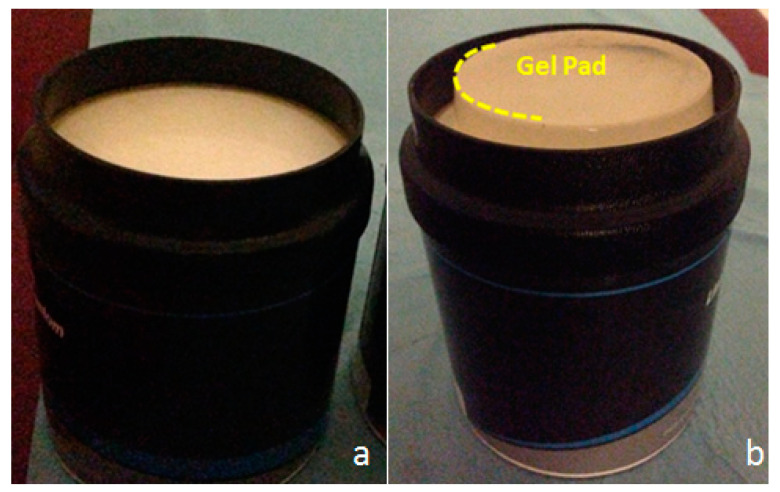
Phantom model 039 “Shear Wave Liver Fibrosis Phantom” (CIRS, Norfolk, VA). (**a**) One example from the series of four homogeneous phantoms of different stiffness (3.5, 11.4, 28.6, and 44 kPa). (**b**) Modified depth: 2 cm gel pad added to measure at 9 cm depth avoiding “edge effects”.

Two sets of phantoms were used to assess both interoperator variability on stiffness measurements and the impact of depth on these measurements. The first phantom, model 049 “Elasticity Phantom—Spherical target map” (Computerized Imaging Reference Systems Inc, Norfolk, VA, USA) provided a set of reference standards to perform stiffness measurements. As illustrated in Figure 1, the phantom consisted of eight Zerdine inclusions, i.e., four inclusions of 10 mm diameter located 15 mm deep with centers separated by 30 mm, and four inclusions of 20 mm diameter located 35 mm deep with centers separated by 30 mm. Each pair of inclusions, which consisted of one inclusion of 10 mm diameter and one inclusion of 20 mm diameter, was calibrated by the manufacturer with different stiffness types as follows: Type I, 8 kPa; Type II, 14 kPa; Type III, 45 kPa; and Type IV, 80 kPa. The second set of four phantoms, model 039 “Shear Wave Liver Fibrosis Phantom” (Computerized Imaging Reference Systems Inc, Norfolk, VA, USA), as illustrated in Figure 2a, was used to assess the impact of depth on the stiffness measurements. These phantoms were made of Zerdine and had cylindrical homogeneous forms, mimicking normal and fibrotic hepatic echo structures. The calibrated phantoms had respective stiffness values of 3.5, 11.4, 28.6, and 44 kPa.

The first phantom was used to assess interoperator variability based on the operators’ experience with ultrasound. Five operators had various experience with the use of classical ultrasound probe, whereas none of them had prior experience with stiffness measurements using UBE. Each operator performed 10 measurements per inclusion, starting with the biggest and the stiffest inclusion, for a total of 80 measurements. Measurements of inclusion size were performed using region of interest (ROI) located at the center of each inclusion. The influence of the size of the inclusions on the measured stiffness was evaluated prior to evaluation of interoperator variability. 

The robustness of UBE was first evaluated through the assessment of reproducibility, repeatability, and variability regarding the influence of the size of the inclusion on the measured stiffness, then according to inclusion type. The influence of the size of the inclusion was assessed by comparing the measured stiffness of the inclusions of 10 and 20 mm for each calibrated type. For every inclusion, the percent bias, i.e., the difference between measured and expected calibrated stiffness divided by the expected stiffness, was computed to evaluate the quality of the measured stiffness. Then, the influence of the operator on the measured stiffness was assessed by computing the coefficient of variation (CV), i.e., the ratio of the standard deviation on the mean of the measured stiffness, between operators. 

The second set of phantoms was used to evaluate the influence of depth on the measured stiffness value. For each of the four phantoms, 10 measurements were made at 3, 5, 7, and 9 cm depth. The phantom was then modified, adding a 2-cm gel pad, to perform 10 measurements at a depth called “9 cm modified” (Figure 2b). This experimental setup was designed to overcome any “edge effects” on measurements due to the proximity of the bottom of the phantom, with previous measurements at 9 cm. Measurements were performed using an ROI of 20 mm, with depth representing the distance between the center of the ROI and the surface of the phantom.

### 2.3. In Vivo Study: Healthy Volunteer Livers

#### Impact of Depth and Acquisition Protocol on Hepatic Segment Stiffness Measurements

Ten healthy volunteer livers were assessed by two radiologists who performed 10 measurements of stiffness per hepatic segment. These measurements allowed assessment of operator influence on the measurements and impact of the acquisition protocol on the results. The acquisition protocol was left to the free judgment of the operator and was carried out blindly regarding depth of the measurements, the approach (intercostal, recurrent, or direct), and ROI positioning. Measurements were performed with ROI ≥ 10 mm and ROI ≤ 20 mm. Depth was defined as the distance between the volunteer’s skin and the ROI’s center. The protocol also allowed volunteers to breathe normally. One parameter that was reported to influence stiffness measurements was the corpulence of the volunteers. The volunteers were then divided into three distinct body mass index (BMI) groups, i.e., Group 1: BMI < 22; Group 2: 22 ≤ BMI ≤ 25; Group 3: BMI > 25.

### 2.4. Evaluation of Texture Feature Variability

**Figure 3:**
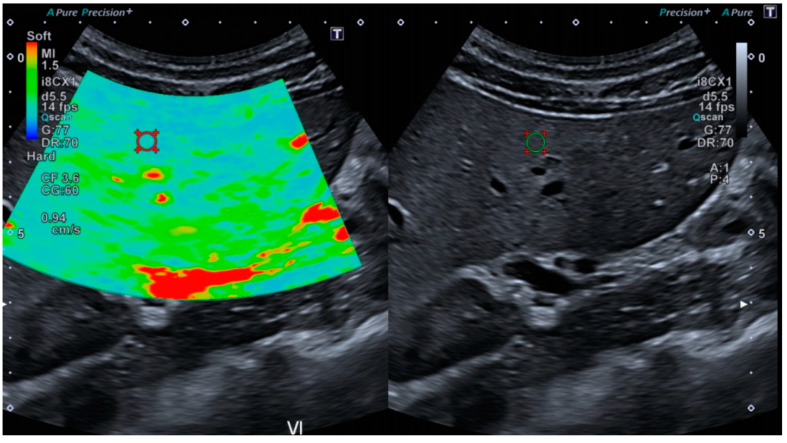
Stiffness map (left) and B-mode image (right) with their respective ROIs of 20 mm diameter used for texture analysis.

The variability of texture features was assessed using images acquired from one healthy volunteer liver. Each image was comprised of a stiffness map and a B-mode image. For each hepatic segment, 10 images were collected for a total of 80 images. Measurements were performed on ROIs of 20 mm, which were placed manually at the same anatomical location on B-mode images and on stiffness maps, as illustrated in Figure 3, resulting in 160 ROIs. The acquisition protocol was left to the free judgment of the operator and was carried out blindly regarding depth of the measurements, the approach (intercostal, recurrent, or direct), and ROI positioning. A total of 35 texture features were extracted from each ROI using LIFEx [13], including four features from the first statistical order (skewness, kurtosis, energy, entropy), 31 from the second statistical order, six from the gray-level co-occurrence matrix (GLCM) (contrast, correlation, dissimilarity, energy, entropy, homogeneity), 11 from the gray-level run length matrix (GLRLM) (GLNU, HGRE, LGRE, LRE, LRHGE, LRLGE, RLNU, RP, SRE, SRHGE, SRLGE), three from the neighborhood gray-level difference matrix (NGLDM) (busyness, coarseness, contrast), and 11 from the gray-level zone length matrix (GLZLM) (GLNU, HGZE, LGZE, LZE, LZHGE, SZE, SZHGE, SZLGE, ZLNU, ZP). Parameters for feature extraction were for B-mode images, as follows: Number of gray levels, 256; min-bound, 0; max-bound, 256; distance with neighbors for the GLCM, 1. Parameters for feature extraction with stiffness maps were as follows: Number of gray levels, 64; min-bound, 0; max-bound, 64; distance with neighbors for the GLCM, 1. For more details regarding the computation of texture features, interested readers are directed to LIFEx documentation. For each segment, the CV of all features was evaluated, and a feature was considered stable for a segment with a CV inferior to 15%.

## 3. Results

### 3.1. Robustness Evaluation Regarding Size and Type of Inclusions

**Figure 4:**
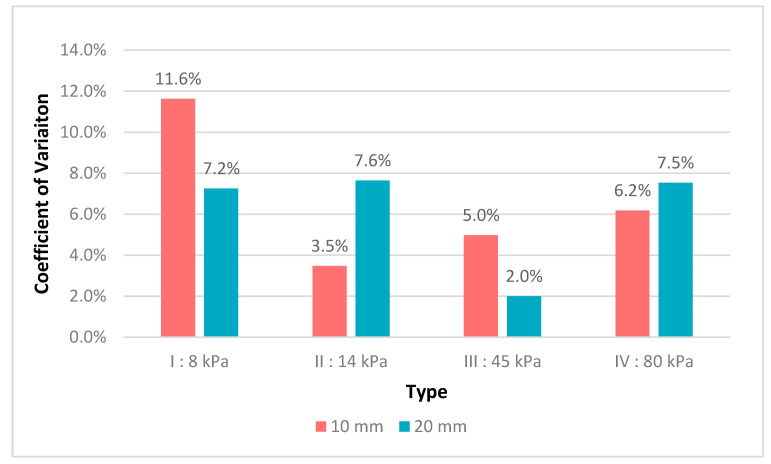
Coefficient of variation for measured stiffness between operators according to type and size of inclusions.

**Figure 5:**
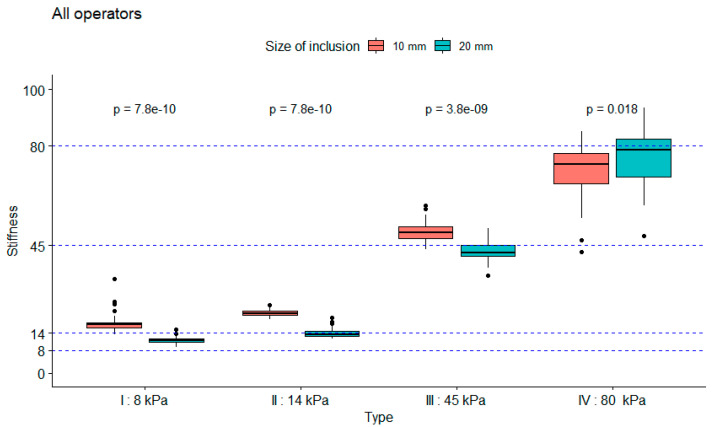
Measured stiffness for all operators, first by type of calibrated inclusion from 8 to 80 kPa, then by inclusion size. Dashed lines represent the expected stiffness for every type of inclusion.

**Figure 6:**
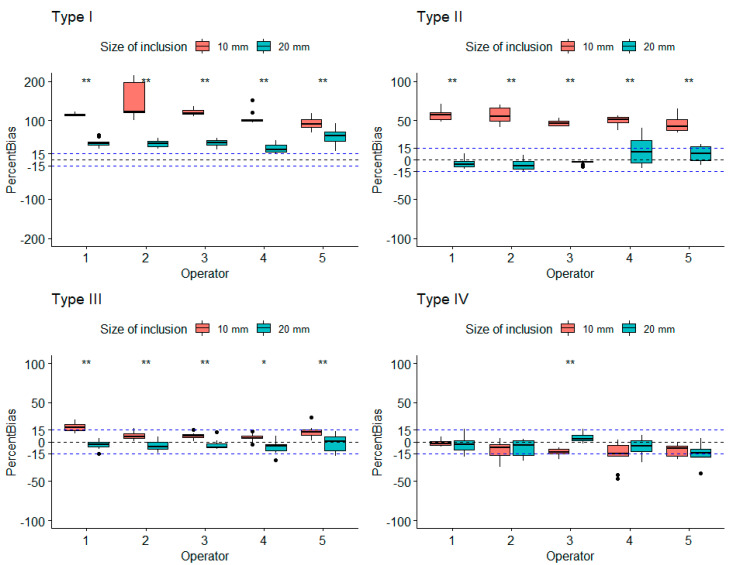
Percent bias of every inclusion for every operator. Dotted lines represent +/-15% indication. Types: I, 8 kPa; II, 14 kPa; III, 45 kPa; IV, 80 kPa.

Measurements were performed by five operators using the first phantom to assess the influence of the size of the inclusions, the influence of the calibrated stiffness, and the influence of the operator on the measured stiffness. The influence of the size of the inclusions was assessed for every type of inclusion. As seen in Figure 4, the coefficient of variability for the mean measured stiffness of every operator was inferior to 12%, which was true for every stiffness-calibrated type of phantom and for all inclusions of 10 and 20 mm. Figure 5, which shows the stiffness measurements according to type and size of inclusions performed by all operators, illustrates the measurement accuracy regarding the expected stiffness at constant inclusion depth and size. These measurements were more accurate with increasing stiffness, and there was a statistically significant difference between measurements performed on inclusions of 10 mm versus inclusions of 20 mm, with measurements on inclusions of 20 mm shown to be more accurate. Both Figure 5 and Figure 6 present that the difference in the measured stiffness according to inclusion size reduced with increasing stiffness. Figure 6 also illustrates that these differences were not operator dependent.

### 3.2. Influence of Depth on Measured Stiffness

**Figure 7:**
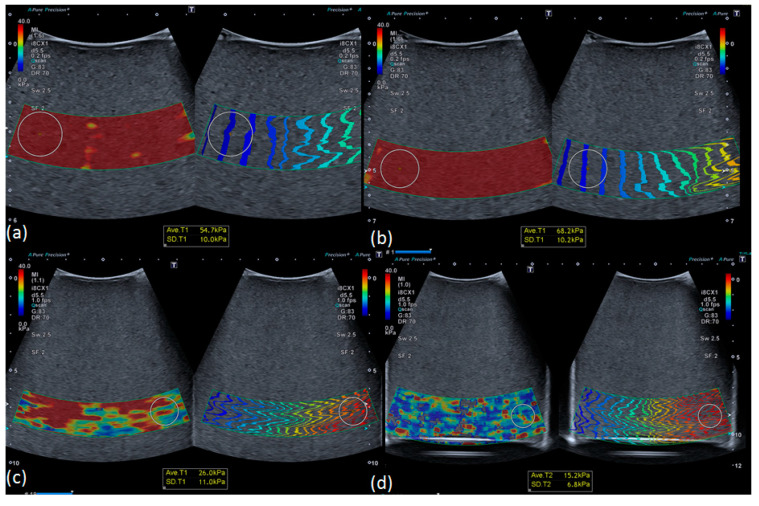
Stiffness maps obtained from 44 kPa phantom at different depths: (**a**) 3 cm, (**b**) 5 cm, (**c**) 7 cm, (**d**) 9 cm. Stiffness maps were homogeneous at 3 and 5 cm; heterogeneity increased as depth increased.

**Figure 8:**
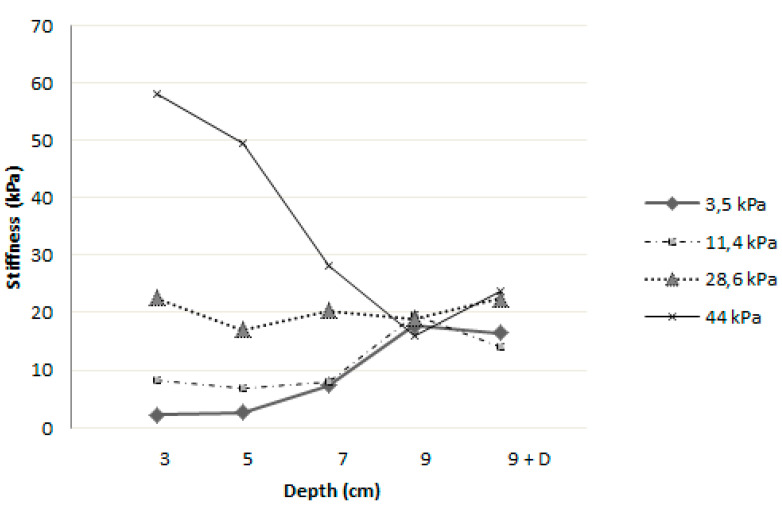
Mean stiffness measurements at various depths for each homogeneous phantom of different calibrated stiffness.

**Figure 9:**
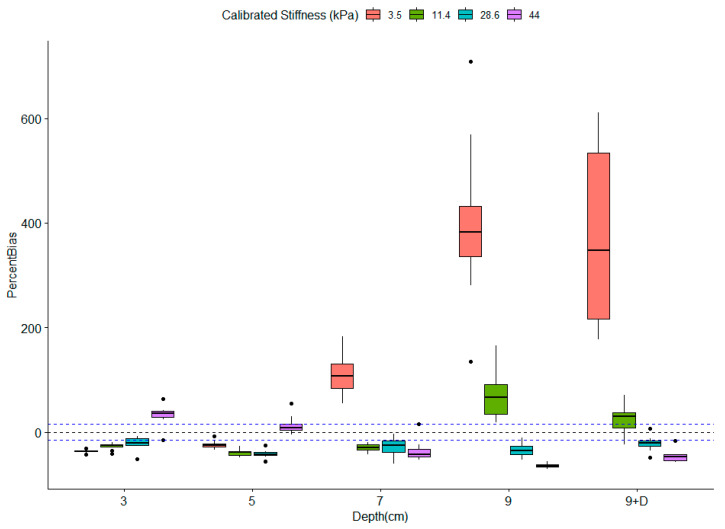
Percent bias (%) for measurements performed on the homogenous phantom series. Percent bias was computed for each series of 10 measurements performed at various depths for each phantom.

The second set of phantoms was used to evaluate the influence of depth on the stiffness measurements. Qualitative analysis of one phantom was performed in order to compare homogeneity of the stiffness maps. Figure 7 illustrates that the stiffness measurements were limited by depth. Indeed, the colored stiffness map showed that heterogeneity increased with depth, suggesting a loss of measurement accuracy. Ten measurements were further performed at each depth (3, 5, 7, 9, and “9 cm modified”) for each phantom of different stiffness (3.5, 11.4, 28.6, and 44 kPa). The averages of each series of 10 measurements were plotted and can be seen in Figure 8. These measurements indicated a convergence of stiffness measurements at the depth of 9 cm, regardless of the theoretical calibrated stiffness. Moreover, as illustrated in Figure 9, the measurements became less accurate with increasing depth and decreasing stiffness, as demonstrated in the previous section. Moreover, for stiffness above 10 kPa, measurements showed relatively low bias and could therefore be trusted up to a depth of 7 cm. 

**Figure 10:**
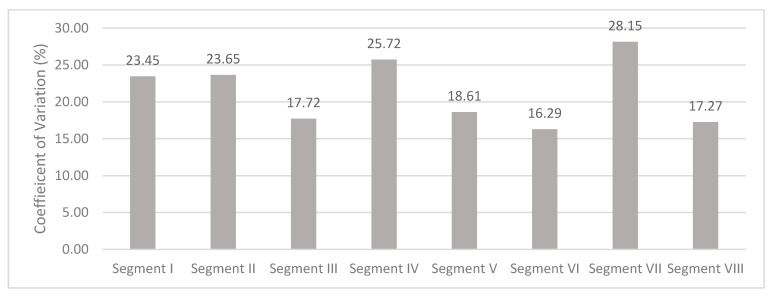
Mean of coefficient of variation (CV) for all stiffness measurements obtained from healthy livers for each hepatic segment.

**Figure 11:**
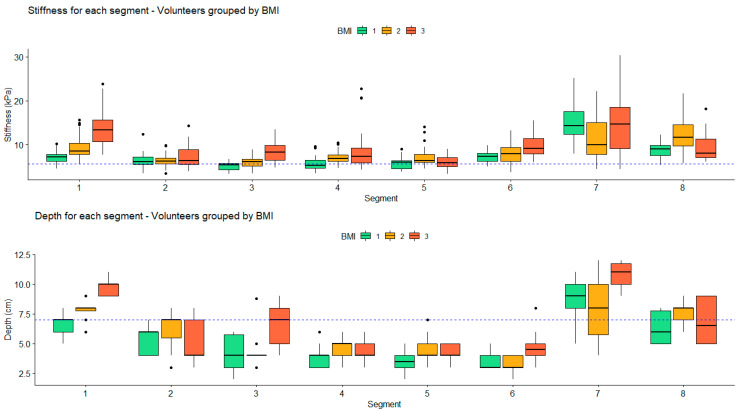
Stiffness measurements and depths at which they were performed for each segment. Results were grouped by volunteers’ BMI index. Dashed lines represent the maximum theoretical value (5 kPa) of nonpathological hepatic segments for the stiffness chart and the maximum depth at which values could be considered accurate based on in vivo results.

On healthy volunteer livers, stiffness measurements could be made on all segments of the liver; CVs are reported in Figure 10. All segments exhibited CVs superior to 15%, but segments III, V, VI, and VIII had CVs inferior to 20%. The values of stiffness and depth were grouped regarding the BMI index of the volunteers and are reported in Figure 11. Overall, there were no significant differences between the BMI groups, both for stiffness and depth. However, a trend was observed indicating that the deepest segments showed increased stiffness compared to shallower segments. Particularly, segments VII and VIII with no distinction between BMI groups and segment I for BMI groups 2 and 3 showed high dispersion of stiffness values up to a factor of three between the minimum and maximum values above 15 kPa. Stiffness measurements were obtained using the ROI located at an average depth of less than 7 cm for segments II, IV, V, and VI, with no distinction between the BMI groups. For segment I, the mean depth was 7 cm for BMI group 1, and for segment III the mean depth was 7 cm for BMI group 3, which was above the other groups in both segments. For segment VIII, the mean depth was below 7 cm for BMI groups 1 and 3, but above for BMI group 2. 

The interoperator variability was also evaluated. All tests suggested that there was no operator influence on the measurements (*p* ≈ 0.5). The impact of the BMI on measurement depth and variability was also assessed, but no statistically significant results were observed.

### 3.3. Robustness of Texture Features in Healthy Livers

On one healthy liver, the variability of 35 texture features was assessed on each hepatic segment; these results are presented in Table 1. Among these features extracted from anatomical images, eight were stable (CV ≤ 15%) in all eight hepatic segments, namely, SRE, RP, correlation, entropy (first and second statistical order), RLNU, SZE, and homogeneity. The mean CVs for these features (as the mean of CVs of the eight hepatic segments) were in the range of (2%, 10%). Moreover, LRE, ZP, GLNU, and dissimilarity were stable, respectively, in 7, 6, 5, and 4 segments, with mean CVs in the range of (11%, 15%). Among the features extracted from the stiffness maps, none demonstrated stability over the eight segments. However, homogeneity and correlation were stable in 6 and 5 segments, respectively, with mean CVs of 11% and 15%.

## 4. Discussion

With the aim of assessing the accuracy and variability of the UBE technique in all hepatic segments, measurements were performed in vitro and in healthy volunteers, up to a maximum depth of 9 cm. Measurements were reproducible in vitro for all inclusions from 8 to 80 kPa, with a CV of less than 12%. Thus, at a maximum depth of 3.5 cm, the in vitro results suggested that operator experience had no influence on stiffness measurements. This finding contradicted Carlsen et al., who demonstrated significant influence by the operator in his phantom study, explained by a better ability to discriminate inclusions [14]. The influence of the size of inclusions was also analyzed, with results indicating that stiffness values measured in the largest inclusions using an ROI of 20 mm were more accurate compared to the smallest inclusions of 10 mm, which demonstrated larger bias (up to 15%) for inclusions of 8 and 14 kPa. This was probably related to the difference in depth between the two inclusions (15 mm and 35 mm for inclusions of 10 mm and 20 mm, respectively), as it is known that the UBE technique of measurement is highly variable at low depths [15]. It is difficult to conclude on the influence of the size of the ROI, because too few studies reported the impact of the size of the ROI; this either represented an exclusion criterion when the tumor’s largest axis was inferior to 10 mm [12], an inclusion criterion when the largest tumor axis was superior to 10 mm [11], or the ROI comprised the whole tumor and the peripheral or stiffest areas were measurements using ROIs that were 10 mm in diameter [9]. Thus, the influence of the size of the ROI and its position (e.g., whole tumor, periphery, stiffest area) should be further studied.

We confirmed that in UBE depth has an influence on elasticity and stiffness measurements, with a cutoff of 7 cm. The depth cutoff varies in the literature from 3–5 cm [16,17] to 6 m [18]. 

In this in vitro study, we observed three different trends in stiffness values. In the case of low stiffnesses, at 3.5 kPa and 11.4 kPa, the measurements were slightly overestimated with increasing depth. This observation was also made in healthy volunteers and previously reported in the literature [19,20]. Huang et al. showed that the average measured rigidity was greater at depths beyond 5 cm with a degraded image success rate in 137 subjects with healthy livers. Choong et al [12] explained that this reduction in measurement reliability was possibly due to fat tissue composition and the presence of several interfaces in the liver parenchyma. Wang stated in his study of 118 healthy volunteers that a lower image success rate with pixels containing no information appeared at depths beyond 5 cm and, as in our study, the mapping was obviously disordered and not uniform or completely different between consecutive measurements. This was probably related to the attenuation of the shear wave by the thickness of the tissue, which can be evaluated according to the obtained heterogeneous stiffness map. For intermediate stiffnesses at 28.6 kPa, no depth influence was observed with good repeatability, consistent with what Wang et al. and Yamanaka et al. [15,19] observed using their homogeneous phantoms of 22.8 kPa and 25 kPa, respectively. Finally, for high-rigidity values, there was a strong dependence on the measurements of the depth. A significant overestimation of stiffness was observed before 5 cm depth, whereas it converged with the other measurements at 9 cm depth, thus proving to be underestimated. This observation was not previously mentioned in the literature but raises the question of the reliability of the measurement of malignant lesions on segments of the liver with values above 20 kPa. 

Practically, these findings imply that the possible use of elastography for monitoring hepatic lesions under treatment should correlate the depth of the measurement with the stiffness of the lesion itself and of the adjacent hepatic parenchyma. This could present a limitation in this particular use, considering that the literature data for liver metastasis, for example, are very heterogeneous. The mean rigidity of liver metastases ranged from 29.5 kPa [9] to 90 kPa [10], with mean surrounding parenchyma values of 5.8 to 11.8 kPa, depending on whether or not the primary tumor was of hepatic origin and the underlying hepatic condition.

Huang et al. evaluated the rigidity of healthy livers on six segments, namely, II/III, IV, V, VI, VII, and VIII, showing that they were all accessible with image success rates of only 24% and 33%, respectively, for the deepest segments VII and II/III. In our study on healthy volunteers, it was possible to evaluate the stiffness within all hepatic segments with maximum success rate, despite depths beyond 7 cm in segments I, VII, and VIII. However, the CV was above 20% in the deepest segments (I, II, IV, and VII). These CVs were much higher than those reported by Huang et al. [20], except for segment VII (36%). These differences were possibly related to the techniques used. 

Some parameters, such as BMI, are known to affect stiffness measurements, which may also interfere with evaluation of the therapeutic response [21]. In healthy subjects, analysis of the results per segment for each BMI group regarding the CV, the depth, and the measured stiffness showed that segments III, V, and VI were suitable for liver stiffness evaluation independent of patient BMI. Moreover, some segments were also suitable depending on the BMI group, e.g., segments I and VIII for BMI group 1, segments II and IV for BMI group 2, and segment VIII for BMI group 3. Stiffness measurements were not statistically different regarding the BMI groups, and no trend was observed. This lack of significance could be explained by the small number of subjects. 

Texture analysis was performed on 20-mm ROIs in every hepatic segment and the robustness of each feature was evaluated. Results showed that, using B-mode or gray-scale images, 12 features presented low variability regardless of the evaluated hepatic segment. In contrast, only two features were stable when extracted from the stiffness maps, and not for every segment. Whereas these features were expected to be more reliable and less variable when evaluated with parametric maps (2D–SWE elastograms), the inhomogeneous nature of the produced maps seemed to be an obstacle for the quantification of reliable texture features. Jian et al. [22] demonstrated that combining texture with elastography on liver fibrosis was of interest due its quantitative advantage. Feature contrast and homogeneity, which demonstrated high diagnostic efficiency in all stages of liver fibrosis, were not considered to be stable enough for the present study. The robustness results of these features extracted from the stiffness maps should also be evaluated with hepatic lesions to assess differences between these and healthy livers. However, the features considered to be stable in this study, particularly with B-mode images, should be evaluated longitudinally to assess if these same features would demonstrate variation during the course of treatment when evaluated over liver lesions. Previous studies reported variable reproducibility results using different imaging modalities. Caramella et al. [23] assessed 34 texture features, computed using eight identical Computed Tomography (CT) acquisitions on a homemade phantom using the same software as in the present study. They observed CVs in a similar range, with a maximum of 18%. Carles et al. [24] assessed the variation of eight texture features computed from Positron Emission Tomography (PET) scan with a maximum CV of 25% over three measurements. Moreover, Abe et al. [25] evaluated the influence of multiple acquisition parameters, such as the dynamic range, focal position, and depth. In the present study, the influence of such parameters, especially depth, was not assessed in regard to texture feature variability, as they were imposed in vitro according to the location of the hepatic segments. Alongside the demonstrated reliability in this study, demonstrating variations during treatment would allow these features to be considered as potential imaging biomarkers, as stated by the imaging biomarker consensus.

## 5. Conclusions

Two quantitative approaches were evaluated regarding their robustness with the aim to further investigate liver lesions.

Measurements of liver stiffness using 2D–SWE can be performed in a reliable and reproducible manner on hepatic segments II to V, as long as the measurements are performed at a depth inferior to 7 cm. The values btained in segments I, VII, and VIII were variable according to the depth of measurement. Texture features were extracted to assess their robustness on a healthy liver. A set of features was demonstrated as stable regardless of the considered hepatic segment when evaluated using B-mode images, whereas few features evaluated using stiffness maps were stable only for some hepatic segments. 

Ultrasound-based elastography and texture analysis of B-mode imaging should be further evaluated to assess tumor responses to treatment.

## Figures and Tables

**Table 1:** Evaluation of the coefficient of variation for texture features extracted from every hepatic segment using one healthy volunteer’s liver, using B-mode and a stiffness map.

Feature	Number of Segments with CV ≤ 15% B-Mode/Stiffness Map	Mean CV B-Mode/Stiffness Map
SRE	8	2%
RP	8	3%
Correlation	8/5	3%/15%
Entropy (GLCM)	8	6%
Entropy (histogram)	8	6%
RLNU	8	7%
SZE	8	8%
Homogeneity	8/6	10%/11%
LRE	7	11%
ZP	6	11%
GLNU	5	15%
Dissimilarity	4	15%

## Abbreviations

GLNU	Gray Level Non-Uniformity for run
HGRE	High Gray-level Run Emphasis
LGRE	Low Gray-level Run Emphasis
LRE	Long Run Emphasis
LRHGE	Long Run High Gray-Level Emphasis
LRLGE	Long Run Low Gray-level Emphasis
RLNU	Run Length Non-Uniformity
RP	Run Percentage
SRE	Short Run Emphasis
SRHGE	Short Run High Gray-level Emphasis
SRLGE	Short Run Low Gray-level Emphasis
HGZE	High Gray-level Zone Emphasis
LGZE	Low Gray-level Zone Emphasis
LZE	Long Zone Emphasis
LZHGE	Long Zone High Gray-Level Emphasis
SZE	Short Zone Emphasis
SZHGE	Short Zone High Gray-Level Emphasis
SZLGE	Short Zone Low Gray-Level Emphasis
ZLNU	Zone Length Non-Uniformity
ZP	Zone Percentage
